# Integrating Genetic Services in the Philippine Public Health Delivery System: The Value of Networks

**DOI:** 10.3390/genes15060780

**Published:** 2024-06-13

**Authors:** Carmencita D. Padilla, Michelle E. Abadingo, Ebner Bon G. Maceda, Maria Melanie Liberty B. Alcausin

**Affiliations:** 1 Department of Pediatrics, College of Medicine, University of the Philippines Manila, Pedro Gil St., Ermita, Manila 1000, Philippines; meabadingo@up.edu.ph (M.E.A.); egmaceda@up.edu.ph (E.B.G.M.); mbalcausin@up.edu.ph (M.M.L.B.A.); 2 Institute of Human Genetics, National Institutes of Health, University of the Philippines Manila, Pedro Gil St., Ermita, Manila 1000, Philippines; 3 Newborn Screening Reference Center, National Institutes of Health, University of the Philippines Manila, Pedro Gil St., Ermita, Manila 1000, Philippines

**Keywords:** genetics, newborn screening, birth defects, rare diseases, genetic counseling, telegenetics

## Abstract

The delivery of genetic services in developing countries is faced with significant challenges, despite medical and technological advances globally. The Philippines, being an archipelago, faces even more challenges, with significant disparities in access to healthcare, and tertiary medical centers and specialists being concentrated in the major cities. The utilization of different networks for the integration of genetic services in the existing public health delivery system has been valuable. Using the well-established network of the national newborn screening program, genetic services have been successfully integrated into the delivery of healthcare, even at the grassroot level. Equitable access to healthcare, including genetic services, was highlighted and supported by the enactment of the Rare Disease Law in 2016. The support of the academe to assure the sustainability of services was evident in the establishment of a genetic counseling program to augment the work of a handful of clinical geneticists. Professional societies and support groups have been instrumental in identifying genetic conditions to be prioritized and lobbying for increased public awareness, leading to national programs and policies. This paper primarily discusses the value of networks in the delivery of genetic services, specifically newborn screening, programs for rare diseases, birth defects, and genetic counseling.

## 1. Introduction

The completion of the Human Genome Project was marked by opportunities for improving population health with the utility of genomics [1]. High-Income Countries (HICs) and Middle-Income Countries (MICs) readily benefited, with sufficient support given to expand the infrastructure for genetic services, impacting public health policies and service delivery, as well as healthcare and social services practices. However, there are barriers to the development of genomic medicine in Low-and-Middle-Income Countries (LMICs) due to a lack of infrastructure, research funding, trained personnel, and specialty healthcare [2,3,4,5,6]. The World Health Organization (WHO) developed guidance for LMIC healthcare delivery in accordance with the *2008–2013 Action Plan for the Global Strategy for the Prevention and Control of Noncommunicable Diseases* (NCDs), with the goal of preventing congenital disorders and genetic diseases at the population level and, at the same time, providing genetics services (diagnosis and counseling) for individuals and families in the community [7,8]. National programs and policies on preventing congenital disorders and minimizing the impact of genetic diseases support the United Nations Sustainable Development Goal (SGD) 3 that focuses on good health and well-being, with one of its success indicators being the end of preventable deaths of newborns and children under 5 years of age [9].

In the past 2 decades, collaborations have grown between countries with developed and developing economies in certain areas of genomic medicine (i.e., training, knowledge transfer, multicenter projects). Research networks have also provided benefits for developed countries, especially with rare diseases having unique clinical features in well-defined populations [1]. Advances in genetic and genomic technology have expanded and improved healthcare services in LMICs in the Asia–Pacific region [3,6].

### About the Philippines and Health Statistics

The geography of the Philippines, an archipelago with 7600+ islands covering 300,000 km^2^, challenges the delivery of basic health services to the Filipino population. In 2023, the population was 117+ M, and it was the 13th most populous country in the world. The Philippines included 1.46% of the total world population, with a population density of 394 per km^2^. The majority of the population (52.9%) reside in rural areas. The Philippines’ annual growth rate is around 1.57%, with a crude birth rate and crude death rate (per 1000 population) of 22.17 and 6.32, respectively. The median age of Filipinos is 25.0 years, with an average life expectancy of 70.4 years for both genders (73.6 years for women, 67.4 years for men). The maternal mortality ratio (MMR) remains high at 78 per 100,000 live births. Infant mortality is 22 deaths per 1000 live births, with a neonatal mortality rate of 15 deaths per 1000 live births [10,11,12].

This report presents some of the strategies that have been successful in integrating genetic services into the Philippine public health delivery system, with a focus on developing and utilizing various healthcare service delivery networks.

## 2. Program Networks for Genetic Services

### 2.1. Newborn Screening Networks

The Philippine newborn screening (NBS) program was introduced in 1996 as a pilot study in 24 hospitals in Metro Manila, and now covers over 7100 hospitals/birthing centers. From an initial NBS panel of 5 conditions, i.e., congenital hypothyroidism (CH), congenital adrenal hyperplasia (CAH), phenylketonuria (PKU), homocystinuria (HCY), and galactosemia (GAL), the screening panel now includes 29 conditions (CH, CAH, glucose-6-phosphate dehydrogenase deficiency [G6PDD], organic acid disorders, amino acid disorders, fatty acid oxidation disorders, urea cycle disorders, galactosemia, hemoglobinopathies, biotinidase deficiency, and cystic fibrosis) [13,14,15]. The vast majority of the included disorders are genetic conditions. Biochemical markers are used for initial screening and confirmatory testing, which include both biochemical and genetic testing. Currently, molecular testing is included as a confirmatory test in the algorithm for fatty acid oxidation defects (FAODs) and hemoglobin disorders. The NSRC is responsible for the national testing database and case registries. The program is currently integrating the FAOD and hemoglobin variants in the program database. Gene panels and targeted gene sequencing are commonly used. Whole-exome sequencing and whole-genome sequencing are occasionally utilized. There is an ongoing initiative to sequence Filipino genomes to serve as a reference sequence.

National and local policies have combined to contribute to increased NBS national coverage. A national law [16,17] enacted in 2004 included the following major highlights: (1) the Department of Health (DOH) is the lead government agency; (2) NBS is a covered benefit of the Philippine Health Insurance Corporation (PHIC); (3) an NBS Reference Center (NSRC) at the University of the Philippines Manila—National Institutes of Health (NIH) exists as the technical implementation partner of DOH; (4) health workers are required to inform parents or legal guardians of the benefit of NBS; and (5) NBS service provision is included in the DOH licensure and accreditation requirements for birthing facilities. Additionally, local government units (LGUs) have issued various resolutions endorsing the law and supporting the accompanying fee collection, among other actions [18].

As of December 2023, the NBS program covers 94.6% of the 1.2 M annual births in the Philippines [19]. Considering the archipelagic nature of the country, a major contributor to accomplishing this coverage rate has been the use of various established networks to support the components of the NBS system: the education of health professionals, parents, and family members; NBS specimen collection and submission; patient tracking and recall for confirmation; and the long-term care and monitoring of newborns with confirmed diagnoses. In situations where the existing network(s) did not adequately address the service delivery challenges, improvement actions were taken to enhance or develop the components.

**Figure 1:**
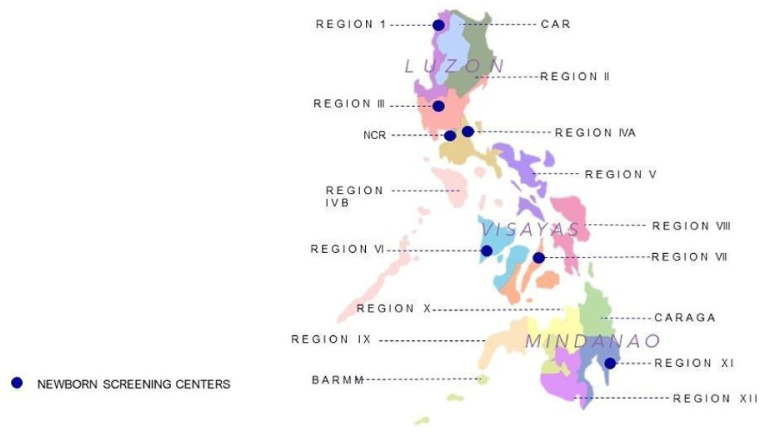
Map of the Philippines showing 3 island groups (Luzon, Visayas, Mindanao), the locations of the 17 administrative regions, and the location of the 7 newborn screening centers.

Figure 1 shows the natural division of the 7600+ islands into three major island-groups—Luzon, Visayas, and Mindanao. Luzon has eight administrative regions, the Visayas has three, and Mindanao has six. There are currently seven newborn screening centers (NSCs) strategically located around the country that provide screening laboratory and follow-up tracking services. Newborn screening facilities (NSFs) are responsible for collecting blood samples via heel prick and drying the blood onto special collection paper (dried blood specimens (DBSs)). DBSs are sent to the NSC assigned for that region to optimize specimen transport and laboratory analysis. To ensure the highest quality of NBS laboratory testing, all NSCs are required to satisfactorily comply with proficiency testing and certification standards that have been established by the DOH/NSRC. This includes participation in international proficiency testing programs and periodic onsite certification reviews. The NSCs form a network of experienced laboratory and follow-up specialists that support each other as needed with training and shared case management, including emergency operations (in the event of an emergency or disaster). Each NSC provides all required laboratory tests and recall/follow activities in accordance with the Philippine Performance Evaluation and Assessment Scheme (PPEAS) defined by the NSRC [16,20].

**Figure 2:**
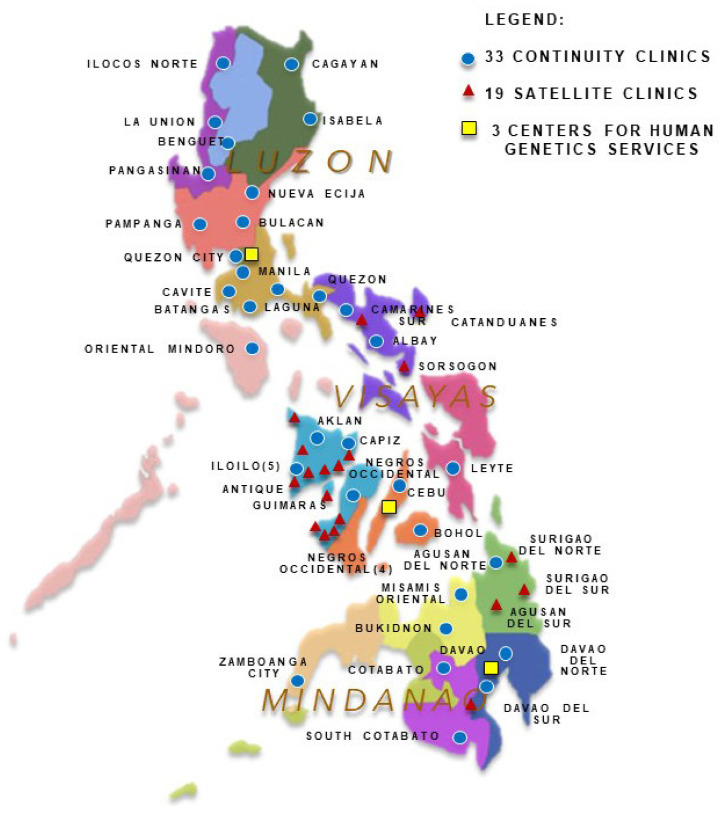
Philippines map showing locations of NBS continuity clinics, satellite clinics, and centers for human genetics services.

Newborns with confirmed diagnoses are referred to newborn screening continuity clinics (NBSCCs), a network of ambulatory clinics based in tertiary hospitals identified by the DOH to be part of the national comprehensive newborn screening system treatment network. NBSCCs are manned by a part-time pediatrician and a full-time nurse, and they facilitate the continuity of care of confirmed patients in their area of coverage [21,22,23]. Satellite clinics have been strategically located to improve tracking and the long-term follow-up of patients in some of the provinces with high numbers of cases (Figure 2). Each clinic is funded by its respective host facility, is manned by a focal nurse and a physician, and is incorporated into Out-Patient Department (OPD) services. They operate at least once a month based on the decking of patients of the host and partner NBSCCs. In the Center for Health Development (CHD) Region 5, the DOH CHD initiates talks with identified hosts (NSFs) and provides support for setting up, such as signages and office equipment, as well financial support for patients.

Guidance for life-long management is provided by the assigned CHGS. As of 2023, there are three CHGSs that provide island-wide services (Luzon, Visayas, and Mindanao) and facilitate comprehensive clinical evaluation, appropriate case management (diagnostic and therapeutic), and genetic counseling services for families or individuals with genetic conditions [13]. Table 1 gives the number of NSFs assigned to the various NSCs, the number of newborns screened, and the number of patients referred to NBSCCs.

**Figure 3:**
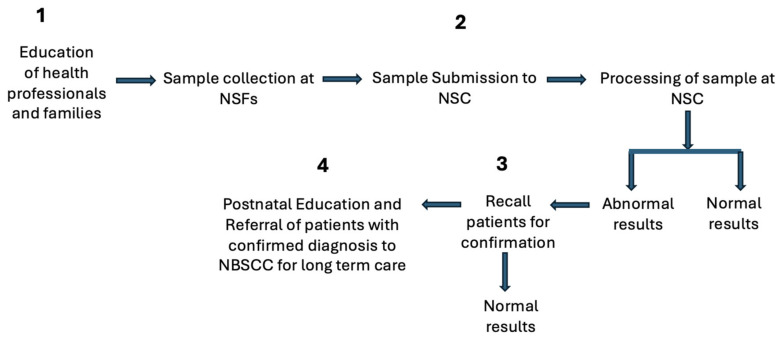
Flow of implementation of the newborn screening program.

The success of the program depends on the support networks. Table 2 lists the defined responsibilities of the support networks. Each is engaged in a necessary and critical support activity, such as specimen collection or follow-up activities (see Figure 3): (1) prenatal and postnatal education; (2) specimen submission; (3) tracking/recall of patients with unsatisfactory, inconsistent, or positive screening results; or (4) the referral of patients with confirmed diagnoses to NBSCCs for long-term care. All of these networks meet regularly to review and discuss best practices, operational challenges, and new ways for the continuous improvement of the program.

### 2.2. Networks for Rare Diseases

The Institute of Human Genetics—National Institutes of Health (IHG-NIH) was instrumental in the preparation, lobbying, and implementation of the Rare Disease Act of 2016. This law provides legislative impetus to improve the access of patients diagnosed to have a rare disease or patients highly suspected of having a rare disease to comprehensive medical care, including drugs and healthcare products, as well as timely health information to help them cope with their condition [24,25].

In December 2017, the Implementing Rules and Regulations were signed by the DOH Secretary [26], shortly followed by the creation of a list of rare diseases by the IHG-NIH (Table 3). This included sixty-five rare diseases, the majority being genetic metabolic disorders, including the disorders being screened by the Philippine newborn screening program. The IRR provides guidance in the implementation of a comprehensive national policy, institutionalizing the system towards the provision of early and sustainable care for persons living with rare diseases (PLWRDs).

It was soon recognized that there was a need to expand the initial list of rare diseases to be more inclusive and comprehensive. The NIH assisted the DOH to accomplish this by heading the development of a Multi-Sectoral Strategic Plan for the Integrated Rare Diseases Management Program (IRDMP) for the period 2022–2026 [27].

#### 2.2.1. Integrated Rare Disease Management Program

*The Integrated Rare Diseases Management Program (IRDMP) Strategic Plan 2022–2026* was the first nationally coordinated effort to address rare diseases in the Philippines. An intersectional plan was developed through consultation sessions that included national government agencies, non-government organizations, medical societies, and patient support groups. The different stakeholders were grouped to discuss eight different areas of support for PLWRDs: (1) financing and funding; (2) communication and information promotion; (3) inclusion and involvement; (4) regulatory and fiscal incentives; (6) research and development studies; (7) the manufacture and importation of orphan drugs and products; and (8) patient support groups. The strategic plan envisioned optimum health outcomes for Filipinos with rare diseases and was anchored by the following principles: timely access; comprehensive, integrative, and sustainable care; evidence-based action and responsiveness; inclusive communication; and enhanced collaboration.

The following six strategic objectives supported the realization of the vision of providing optimum health outcomes for Filipinos with rare disease: (1) integrated comprehensive care for PLWRDs within the public healthcare delivery system; (2) PLWRD access to entitlements and benefits; (3) the creation of health promotion, public information, and education campaigns on rare diseases; (4) the provision of evidence for policy and program planning through research and development; (5) increased availability and access to orphan drugs and products; and (6) financial assistance to PLWRDs.

In addition to RA 10747, the IRMDP was also supported by Republic Act 11223, or the Universal Health Care Act [28], enacted in 2018. This provided a healthcare model that provides all Filipinos access to a comprehensive set of quality, cost-effective, promotive, preventive, curative, rehabilitative, and palliative health services without causing financial hardships, and prioritizes the needs of the population who cannot afford such services. The law ensures that all Filipinos are guaranteed equitable access to quality and affordable healthcare services, and are protected against financial risks.

Included in the strategic plan is the generation of a list of rare diseases developed through collaboration with different medical societies. To assist in decision-making as to which rare diseases were to be included in the expanded list covered by RA 10747, a scoring system was created that included prevalence, impact, diagnosis, and treatment. The IRMDP team conducted online meetings with the representatives of different medical societies/subspecialty sections: 16 affiliated with the Philippine Pediatric Society (PPS): 12 with the Philippine Obstetrical and Gynecological Society (POGS); 7 with the Philippine College of Physicians (PCP); and 6 with the Philippine College of Surgeons (PCS). To ensure inclusivity, executive committee members were assigned per society, and they were tasked to send invites to their component subspecialty societies. Each subspecialty society was tasked to submit the top five rare diseases encountered in their subspecialty.

An adjudication was accomplished with the following objectives: (1) to finalize the list of rare diseases; (2) to decide on what to do in case of an overlap in the given conditions; (3) to decide whether a rare disease submitted was approved, conditionally approved, or did not qualify; and (4) to resolve other concerns. The adjudication included the executive committees of the four major societies. Out of the 166 diseases submitted, a more inclusive list of 96 additional rare diseases passed the Rare Diseases Scoring System. The collaboration with the different subspecialties allowed for the inclusion of other conditions such as rare cancers, immunologic disorders, neurologic disorders, gastrointestinal disorders, infectious diseases, hematologic disorders, endocrinologic disorders, rheumatologic disorders, gynecologic disorders, vascular disorders, dermatologic disorders, skeletal dysplasias, urogenital disorders, pulmonary disorders, and structural congenital anomalies, among others (Table 4).

#### 2.2.2. Inter-Hospital and Intra-Hospital Networking through Multidisciplinary Clinics

Individuals affected by genetic conditions often require management cutting across many subspecialties. The multi-organ involvement in these conditions necessitates consultations not only with general medicine and clinical genetics departments, but also cardiology, endocrinology, neurology, pulmonology, otorhinolaryngology, ophthalmology, orthopedics, and rehabilitation departments, among others. Allied health professionals, such as dentists, physiotherapists, and occupational therapists, are integral in the management of these individuals with life-long medical needs.

Subspecialists are few, with most practicing in cities where tertiary hospitals are located. Cooperation and communication among health institutions and among subspecialties within the tertiary hospitals has evolved into an effective referral network. Multidisciplinary clinics (MDCs) bring together different subspecialties to provide care for a special group of patients in a single clinic. This is particularly helpful for individuals with limited mobility and for patients from remote areas to be seen in a single day. While convenience in meeting with multiple medical care providers in a single clinic is the primary goal, multidisciplinary clinics also provide venues for the simultaneous review of specialty management plans for individual patients, allowing a more efficient and effective delivery of cohesive and comprehensive care. The following MDCs have been organized by the Division of Clinical and Metabolic Genetics of the Department of Pediatrics, Philippine General Hospital, in coordination with the Institute of Human Genetics, National Institutes of Health, University of the Philippines Manila:

(1) Mucopolysaccharidosis (MPS) Multidisciplinary Clinic—A bi-annual clinic attended by an average of 15 patients per clinic day. It has provided 349 consultations to both new and follow-up patients since it started in 2009 [29]. The most common MPS seen in this clinic is MPS II.
(2) Osteogenesis Imperfecta (OI) Multidisciplinary Clinic—A bi-annual clinic attended by an average of 18 patients per clinic since 2014. Moderate-to-severe OI phenotypes are seen frequently in this MDC.
(3) Skeletal Dysplasia Multidisciplinary Clinic [30]—Initially an annual clinic that opened in 2018, this transitioned to a quarterly clinic in 2024, and is attended by an average of eight patients per clinic. Most patients who attend this clinic have achondroplasia.
(4) Neuromuscular Multidisciplinary Clinic—A quarterly clinic since 2018, initiated by pediatric neurologists at the PGH and attended by 10–15 patients per clinic. The majority have Duchenne muscular dystrophy (DMD).

#### 2.2.3. Patient Support and Volunteer Groups

Patient support groups and volunteer groups are effective allies in raising awareness of rare genetic conditions not only among families with genetic conditions, but also the population at large. Lobbying for better access to quality care, access to health professionals knowledgeable about their conditions, and access to specialized diagnostics and medicine are best accomplished with the individuals affected and their families at the forefront. The Philippine Rare Disease Law is a testament to this. Some of these patient support groups include the Philippine Society for Orphan Disorders; the Down Syndrome Association of the Philippines, Inc.; the Osteogenesis Imperfecta Support Group, Philippines; Big Dreams for Little People, Philippines, Inc.; and Hemophilia Philippines (HAPLOS Community). Advocacy for an increased awareness of birth defect prevention, the importance of folic acid, its inclusion in dietary fortification, and newborn screening have been the focus of the volunteer youth leaders of Health Philippines. Founded in 2009, this group of students from different universities works with their fellow students and the general public using social media and promotional materials for broad outreach.

## 3. Genetic Counseling Networks

The genetic counseling services in the Philippines are offered by both clinical geneticists and genetic counselors. Referrals for genetic counseling are given to prenatal, pediatric, metabolic, neurologic, and cancer patients. Currently, there are nineteen clinical geneticists practicing in the Philippines: three in the Visayas, one in Mindanao, and the rest in Luzon. With the current population of more than 117 million, the clinical-geneticist-to-population-density ratio is 1:6,160,000, which is an improvement from 1:11,750,000 a decade ago [31].

### 3.1. Local Networks

In the last two decades, there have been formal and informal collaborations between US-based geneticists/genetic counselors and local geneticists and clinicians for the delivery of direct genetic services. These collaborations made possible the establishment of a formal Master of Science in Genetic Counseling in the Philippines, aiming to reduce genetics-trained personnel shortages across the country [31].

The Master of Science in Genetic Counseling program at the College of Medicine, University of the Philippines Manila, started in 2011. The creation of the master’s program was in anticipation of the increased demand for genetic services brought about by the expansion of the NBS and the increasing utility of genetics and genomics in several medical specialties [24,32,33,34]. Since 2011, the program has successfully produced 18 genetic counselors, contributing to easier access to genetic counseling services across the country.

The Philippine Society of Genetic Counselors (PSGC) was formally organized in 2021 as the lead professional organization that advances the field of genetic counseling in the country, ensuring that genetics and genomics care are accessible and delivered equitably and safely to all Filipino families. As many healthcare workers are not yet familiar with this service, awareness through regular webinars engaging in various medical specialties have been organized by the society.

Genetic counseling for neurology, cancer, and prenatal patients is practiced on a limited basis. For the neurogenetic condition X-linked dystonia parkinsonism (XDP), the role of the Sunshine Care Foundation for Neurological Care and Research (SCF) in counseling service delivery has been critical. This non-profit NGO based in Roxas City, Capiz, provides genetic counseling and genetic testing for XDP patients [34]. Additionally, multidisciplinary clinics for neuromuscular disorders (NMDs) held at the Philippine General Hospital, organized by the Division of Pediatric Neurology Service, allow families and patients with DMD and other NMDs to access comprehensive and coordinated medical care, including genetic counseling and testing.

Republic Act No. 11215, or the National Integrated Cancer Control Act [35], is aimed at improving and providing integrated care to patients with cancer. The importance of genetic counseling and testing is also highlighted. In a local study, cancer risk perception and screening behavior were noted to be important factors that must be addressed during cancer genetic counseling consultations [36]. With the increasing awareness of oncologists of the value and availability of genetic counseling for cancer patients, the demand and service delivery also increased.

Prenatal genetic counseling services primarily serve to help obstetricians with case management. Furthermore, the termination of pregnancies is not practiced [37]. However, noninvasive prenatal testing (NIPT), which is performed from 10 weeks of pregnancy and involves the measurement of circulating cell-free DNA (cfDNA) in the maternal blood, is already available in the country. In fact, there are at least three laboratories that offer NIPT, with prices ranging from PHP 24,500 to 55,000, or USD 491 to 1103. This can lead to challenges for geneticists/genetic counselors to provide effective, insightful, and helpful counseling [38]. Prenatal diagnosis is therefore the aim so that anticipatory guidance can be provided and parents can prepare for the birth of a child with birth defects [39].

### 3.2. International Networks

The Asia Pacific Society of Human Genetics (APSHG) and the Professional Society of Genetic Counselors in Asia (PSGCA) have been vital in providing continuing education for genetic counselors in the region and locally through meetings where updates are shared, and cases and challenges are discussed [33]. The Asia Pacific Conference on Human Genetics (APCHG) is held every two years, alternating with the APSHG Summer or Autumn School on Human Genetics. In addition to providing updated knowledge on the field, these activities promote networking among clinical geneticists, genetic counselors, and trainees from the Asia–Pacific region. Using various fora where updates in genetics/genomics and genetic counseling are shared to various professionals, including health professionals, health policy makers, legislators, and the general public, the importance of this service is gaining much needed attention.

### 3.3. Telegenetic Counseling

Telehealth or telemedicine already existed as a service prior to the pandemic. Telegenetic counseling uses videoconferencing as another means of providing genetic counseling [40]. Telegenetic counseling is being provided to patients and families with transportation challenges, especially those living in geographically isolated and disadvantaged areas. Its creation has allowed access to quality genetic counseling services across the country. Through the network of the NBS program, which includes the regional offices of the DOH, the NBSCCs, the NSCs, and the Center for Human Genetics Services, genetic counseling and medical evaluations/the management of patients with genetic and metabolic conditions are available. This service, however, comes with both advantages and disadvantages. Telegenetic counseling is advantageous for patients since it decreases travel time and reduces time away from work. Disadvantages, on the other hand, include longer sessions due to unstable internet connections, poor mobile network signals, and an inability to assess nonverbal cues from patients [41]. More efficient counseling has been accomplished through a support network of partners who meet with and assist patients in local clinics using a combination of face-to-face contact and telegenetics.

In a span of 14 months (January 2019–February 2020), a total of 43 patients out of 275 (15%) underwent telegenetic counseling prior to the pandemic. This number increased significantly during the pandemic (March 2020–March 2021), where 519 patients underwent telegenetic counseling. The network of support from the different newborn screening stakeholders increased the availability of this service to a broader reach across the entire country. Various modes of telemedicine were performed, including video call, phone calls, and even social media, particularly Facebook messenger [41].

## 4. Discussion

Population-based services include health promotion, preventive interventions, and health services that reach the entire population of a country. In LMICs, population-based services related to genetics often include multiple subject areas: preconception screening and counseling (e.g., family history, teratogens, folic acid); outreach/public education; prenatal screening and counseling (e.g., Rh incompatibility, maternal serum screening); newborn screening (e.g., bloodspot, hearing, CCHD); childhood screening (e.g., physical examination, medical and family history); and adulthood screening (e.g., breast and bowel cancer screening). [2]. Identified barriers for population-based genetic screening include participants’ psychosocial, attitudinal, and belief-related factors, and providers’ perceived limited clinical utility [42]. In less developed countries, these services are limited.

Prior to the 1990s, genetic services in the Philippines were limited to cytogenetic services at two institutions. Due to the scarcity of formally trained geneticists, the diagnosis of genetic syndromes was a constant challenge. Recognition of the importance of the diagnosis, management, and prevention of complications from heritable conditions led to the establishment of the Institute of Human Genetics at the University of the Philippines Manila in 1999 by the Board of Regents of the University of the Philippines. Before the establishment of the Institute, there were no local statistics on genetic disorders [43]. Through research, the academe provided the data needed by the government to establish national programs to address this neglected issue, beginning with the NBS program. The successful implementation of the NBS was primarily due to a network spanning the public health system from the DOH at the national level to NSFs at the grassroots level, which included significant capacity building contributions from the academe and NGOs.

The most successful healthcare system in the Philippines with some form of integrated genetic services is the national NBS program. With legislative support, this program has provided a template for the formal offering of genetic services. The comprehensive program has integrated health promotion activities at the community level with a dynamic tri-media campaign and various support networks enabling the implementation of the screening system. NBSCCs provide long-term care services to conditions outside of the expanded newborn screening panel. The NBS program is committed to sharing these NBSCCs with patients with rare diseases (not included in the ENBS panel) and birth defects as part of its network support for the DOH. The goal is to have eighty-one active NBSCCs (at least one per province, with the support of satellite clinics).

Through the IRDMP, a comprehensive program for PLWRDs has been initiated. The challenge now is to complete and sustain its operation. Through collaborations among different disciplines, multidisciplinary clinics for select conditions allow more effective and efficient healthcare service delivery. The intent is to replicate this framework in other areas of the country. Collaborations with patient support groups and volunteer groups will be vital for increased awareness.

While the infrastructure has been established, the continuing challenge is the lack of enough geneticists and genetic counselors. There are only 19 clinical geneticists and 10 genetic counselors serving a population of 117 M+ with 1.2 M annual births. Telegenetics has contributed to service availability, but the volume of patients served has been limited. The potential of telegenetics is yet to be fully maximized. The challenges of telegenetics service delivery, including data/patient privacy, organizational readiness, digital maturity, geographical and digital disparities, and integration with traditional medical services, must also be overcome for optimal operation [44].

An awareness of the value of genetics and genomics, the availability and accessibility of genetics services, and the existence of genetic and other rare diseases has been improving over the years through the different networks previously discussed. There is an increasing trend of subspecialists in the Philippines who provide genetic testing options to their patients. At the moment, the majority of tests are sent out to laboratories overseas. As part of multidisciplinary care, patients are referred to clinical geneticists or genetic counselors to facilitate informed decision-making about undergoing a specific gene test. Genetic counseling will give information about a particular test, as well as its benefits, risks, and limitations, and the possible results of the test.

Different patient support and volunteer groups play a critical role in spreading awareness of genetics services at the community (e.g., community health workers) and institutional (e.g., medical genetics programs) levels [45]. Although genetic programs in the Philippines are at different levels of maturity, it is clear that local and international networks and collaborations are vital for the success and sustainability of these.

## 5. Conclusions

The integration of genetic services in existing public health delivery systems entails collaboration in both local and international networks. Experiences and efforts shared through international meetings among regional and global networks are crucial in establishing and improving the quality of genetics services. The lessons learned allow the identification of which services to adopt and improve in local settings. Strong local networks are vital to the delivery of genetic services. Concerted efforts of government and non-government units, professional societies, and the academe are critical to make the genetics service delivery efficient and sustainable. Supported by national legislation, the service delivery foundation is strong for integrating these services into the public health delivery system.

## Figures and Tables

**Table 1:** NSCs, NSFs, number of newborns screened, and number of newborns endorsed to NBSCCs.

NSC	Host	Regions Covered	No. of NSFs *	No. of Newborns Screened *	No. of NBSCCs *	No. of Patients Endorsed to NBSCCs
**LUZON**						
NSC—Northern Luzon	Mariano Marcos Memorial Hospital	I (Ilocos) II (Cagayan Valley)	621	112,145	5	830
NSC—Central Luzon	Angeles University Foundation Medical Center	III (Central Luzon) Cordillera Administrative Region	1155	200,258	4	1681
NSC—NIH	National Institutes of Health, University of the Philippines Manila	NCR (National Capital Region) V (Bicol) IVB (MIMAROPA)	1661	298,776	5	1737
NSC—Southern Luzon	Daniel Mercado Medical Center	IVA (Calabarzon)	1259	189,662	4	1174
**VISAYAS**						
NSC—Central Visayas (Cebu)	Eversley Childs’ Sanitarium and General Hospital	VII (Central Visayas)	526	103,214	2	672
NSC—Visayas (Iloilo)	West Visayas State University Medical Center	VI (Western Visayas) VIII (Easter Visayas)	805	152,294	5	1142
**MINDANAO**						
NSC—Mindanao (Davao)	Southern Philippines Medical Center	IX (Zamboanga) X (Northern Mindanao) XI (Davao) XII (SOCCSKSARGEN) XIII (Caraga) BARMM (Bangsamoro Autonomous Region in Muslim Mindanao	1942	320,922	8	2287
**TOTAL**			**7969**	**1,377,271**	**33**	**9523**

* for the year 2023.

**Table 2:** Networks and their responsibilities in different stages of newborn screening implementation.

Networks	Responsibilities
**Education**	
National Level—Newborn Screening Society of the Philippines, Inc. (NSSPI)	The NSSPI is a non-stock, non-profit organization of health professionals dedicated to the promotion and advancement of newborn screening in the Philippines. Together with the NSRC, it conducts year-round educational activities for professionals and the public. It hosts an annual convention in October every year during the Newborn Screening Awareness Week, as declared by Presidential Proclamation 530. It is attended by physicians, nurses, midwives, and medical technologists. The NSSPI has been hosting annual conventions since 2003.
Regional Level—Centers for Health Development (CHDs)	The CHDs are responsible for the field operations of the various public health programs of the department in each of the 17 regions. They coordinate with LGUs, the DOH partners, in the implementation of various public health programs. A full-time nurse is assigned to the NBS program to assist in region-wide activities to increase the awareness of newborn screening.
Local Level—Provincial, District and Municipal Health Units	Health teams at various levels coordinate with the CHD in organizing awareness programs, especially during the Newborn Screening Awareness Week.
Newborn Screening Reference Center	Oversees the content of educational materials in coordination with the DOH Health Promotion and Communication Services (DOH HPCS).
NSCs	Conducts various awareness and educational activities such as the development of videos, materials, events, and social media promotions.
NBS sample collection and submission	
NSFs	Every NSF has a hospital team consisting of physicians, nurses, and/or midwives to take charge of collecting samples and sending them to their respective NSCs.
**Release of NBS Results and Recall**	
NSC Short-Term Follow-Up Teams	Every NSC has a follow-up team that takes charge of following up all patients with positive screening results. They coordinate with the hospital team in collecting additional samples for confirmation.
CHDs	For patients who cannot be readily contacted, the CHDs assist the NSF in tracking the patient.
**Long-Term Follow-Up**	
NBSCCs	Continuity clinics monitor patients’ compliance to treatment to improve outcomes. The NBSCC teams facilitate online and face-to-face consultation with specialists.
CHDs	CHDs assist in tracking down patients who are not compliant with schedules of follow-up.
Philippine Society of Pediatric Metabolism and Endocrinology	Endocrinologists across the country assist with the long-term care of patients diagnosed with CH and CAH.
CHGS	Provides comprehensive clinical evaluation and laboratory diagnostic services to families or individuals with or at risk of inheritable disease. It also provides support for the Telegenetics Referral System.

**Table 3:** Initial list of rare diseases identified by the Institute of Human Genetics—National Institutes of Health.

Disorders Included in Philippine ENBS Panel	Disorders not included in the Philippine ENBS Panel
*Amino Acid Disorders* 6- Pyruvoyltetrahydropterin synthase deficiency (6-PTPS) Homocystinuria Hyperphenylalaninemia Maple syrup urine disease (MSUD) Methionine adenosyltransferase deficiency (MAT) Phenylketonuria (PKU) Tyrosinemia type I, II, III *Fatty Acid Oxidation Disorders* Carnitine palmitoyltransferase 1 (CPT 1) deficiency Carnitine palmitoyltransferase 2 (CPT 2) deficiency Carnitine-uptake deficiency (CUD) Glutaric aciduria type II (GA 2) Long-chain hydroxyacyl-CoA dehydrogenase deficiency (LCHADD) Medium-chain-Acyl-CoA dehydrogenase deficiency (MCADD) Trifunctional protein (TFP) deficiency Very-long-chain-Acyl-CoA dehydrogenase deficiency (VLCADD) *Organic Acid Disorders* 3-Methylcrotonyl-CoA carboxylase deficiency (3MCC) Beta-Ketothiolase deficiency Glutaric aciduria type I (GA 1) Holocarboxylase/multiple carboxylase deficiency Isovaleric acidemia (IVA) Methylmalonic acidemia (MMA) Propionic acidemia (PA) *Urea Cycle Disorders* Argininosuccinate lyase deficiency Citrullinemia type 1 *Others* Biotinidase deficiency Galactosemia	*Glycogen Storage Diseases and Related Disorders* Glycogen storage disorders Pompe disease *Disorder of Amino Acid Transport at the Cell Membrane* Cystinuria *Hyperphenylalaninemia* Dihydropteridine reductase (DHPR) deficiency *Disorder of Intracellular Triglyceride and Phospholipid Metabolism* Lowe syndrome *Disorders of Metal Transport* Menkes disease Wilson disease *Inborn Errors of Non-Mitochondrial Fatty Acid Metabolism* Refsum disease X-linked adrenoleukodystrophy (X-ALD) *Disorders of Neurotransmission* Succinic semialdehyde dehydrogenase (SSADH) deficiency *Disorders of Sphingolipid Synthesis, Sphingolipidoses, Niemann–Pick Disease Type C and Neuronal Ceroid Lipofuscinoses* Fabry disease Gaucher disease GM1 gangliosidosis Krabbe disease Niemann–Pick disease Neuronal ceroid lipofuscinoses (NCL) Tay–Sachs disease *Mucopolysaccharidoses* Mucopolysaccharidosis (MPS) type 1 MPS 2 MPS 3 MPS 4 MPS 6 Multiple sulfatase deficiency *Mucolipidoses* Mucolipidosis *Disorders of Urea Cycle and Related Enzymes * Citrin deficiency Carbamoyl phosphate synthetase 1 (CPS I) deficiency N-Acetylglutamate synthase (NAGS) deficiency Ornithine transcarbamylase (OTC) deficiency *Organic Acid Disorders* 3-Methylglutaconic aciduria L-2 hydroxyglutaric aciduria *Disorders of Oxidative Phosphorylation* Leigh syndrome Mitochondrial encephalopathy, lactic acidosis and stroke-like episodes (MELAS) Mitochondrial depletion syndrome (MDS) *Inborn Error of Purine Metabolism* Lesch–Nyhan disease

**Table 4:** List of additional rare diseases included from consultation with medical societies.

Rare cancers Acute lymphoblastic leukemia Basal cell nevus syndrome (Gorlin–Goltz syndrome) Bladder cancer Gastric cancer Gastrointestinal stromal tumor (GIST) Glioma Hodgkin lymphoma Laryngeal cancer Malignant melanoma Neuroendocrine tumor, cervix Pancreatic cancer Primary CNS cancer Primary fallopian tube cancer Pseudomyxoma peritonei Rectal gastrointestinal stromal tumor Retinoblastoma Serous ovarian cancer Sertoli–Leydig cell tumor Uterine leiomyosarcoma Uterine sarcoma Vulvar adenoid cystic carcinoma Wilms tumor Rare immunologic disorders Chronic granulomatous disease Evans Syndrome Hyper IgE syndrome/Job’s syndrome IgG4-related disease Immune-mediated inflammatory myopathies Langerhans cell histiocytosis Severe combined immunodeficiency Wiskott–Aldrich syndrome X-linked agammaglobulinemia (XLA) Rare neurologic disorders Congenital central hypoventilation syndrome Menkes disease Neurofibromatosis type 2 Rett syndrome SeSAME syndrome Spinal muscular atrophy Syndromic craniosynostosis X-linked dystonia (lubag) Rare gastrointestinal disorders Achalasia Biliary atresia Choledochal cyst Cloacal malformation (persistent cloaca) Eosinophilic colitis Idiopathic neonatal hepatitis Inflammatory bowel diseases Rare infectious diseases Cat-scratch disease HIV in pregnancy Lyme disease Melioidosis Mucormycosis in pregnancy Mycetoma Yaws (endemic treponematoses)	Rare rheumatologic disorders Juvenile idiopathic arthritis Juvenile systemic sclerosis Polyarteritis nodosa Scleroderma Systemic lupus erythematosus Takayasu arteritis Rare hematologic disorders Aplastic anemia Diamond blackfan anemia Erdheim–Chester disease Hemophilia B Polycythemia vera Rare gynecologic disorders Achard–Thiers syndrome Distal vaginal agenesis Herlyn–Werner–Wunderlich syndrome (OHVIRA) Unicornuate uterus Rare endocrinologic disorders Central diabetes insipidus Congenital adrenal hyperplasia Congenital hyperinsulinism Neonatal diabetes Rare vascular disorders Aneurysms in children Behcet disease DAVF in children Primary systemic vasculitis Rare dermatologic disorders Epidermolysis bullosa Generalized pustular psoriasis Ichthyotic skin disorders Rare pulmonary disorders Idiopathic pulmonary arterial hypertension Interstitial lung disease Rare urogenital disorders Fowler’s syndrome Ochoa syndrome Rare structural congenital anomalies Conjoined twins Exstrophy of the bladder Rare ophthalmologic disorder Primary congenital glaucoma Rare skeletal dysplasias Osteogenesis imperfecta Rhizomelic chondrodysplasia punctata Other rare disorders Juvenile breast hypertrophy Gonadal dysgenesis Hutchinson–Gilford progeria Lowe syndrome Prader–Willi syndrome Smith–Lemli–Opitz syndrome Smith–Magenis syndrome

## Data Availability

The original contributions presented in the study are included in the article, further inquiries can be directed to the corresponding authors.
